# Editorial: Microglia in neurodegenerative diseases

**DOI:** 10.3389/fncel.2024.1473576

**Published:** 2024-09-24

**Authors:** Ting Li, Ana María Espinosa-Oliva

**Affiliations:** ^1^Gansu Key Laboratory of Biomonitoring and Bioremediation for Environmental Pollution, School of Life Sciences, Lanzhou University, Lanzhou, Gansu, China; ^2^Institute of Biomedicine of Seville (IBiS), Hospital Universitario Virgen del Rocío, CSIC, University de Seville, Seville, Spain; ^3^Department of Biochemistry and Molecular Biology, Faculty of Pharmacy, University of Seville, Seville, Spain

**Keywords:** microglia, neurodegenerative diseases, cell interactions, neuroinflammation, *in vivo* imaging tools

Neurodegenerative diseases are a heterogeneous group of disorders that share, among other characteristics, chronicity and progression. These diseases present a significant challenge for medical research due to their high prevalence, leading to substantial morbidity, functional loss, and frequently resulting in death due to poor diagnosis. Currently, there are very few effective treatments, and none that can cure these conditions. Consequently, neurodegenerative diseases, along with mental diseases, contribute significantly to human suffering. Understanding the molecular mechanisms underlying these diseases is crucial for developing new and effective therapies aimed at preventing and/or slowing down the degeneration of nerve cells.

There are various theories that try to elucidate the mechanisms involved in the degeneration of nerve cells, being one of them neuroinflammation, inflammation of neural tissue particularly mediated by glial cells in response to brain infection, damage or entry of substances from the peripheral blood into the CNS. In fact, many neurodegenerative diseases, such as Parkinson's disease (PD), Alzheimer's disease (AD), and Amyotrophic lateral sclerosis (ALS), exhibit markers of inflammation. Therefore, microglia cells, which are key players in the neuroinflammatory process, as well as in a variety of other processes, play a crucial role in the pathogenesis of these diseases.

Microglia are the resident immune cells of the central nervous system (CNS). While they are essential for maintaining tissue homeostasis, an increasing number of studies have shown that microglia are involved in all stages of neurodegenerative diseases and significantly impact disease progression. However, the role of microglia in pathological development remains controversial due to their highly dynamic and plastic nature.

**Figure 1:**
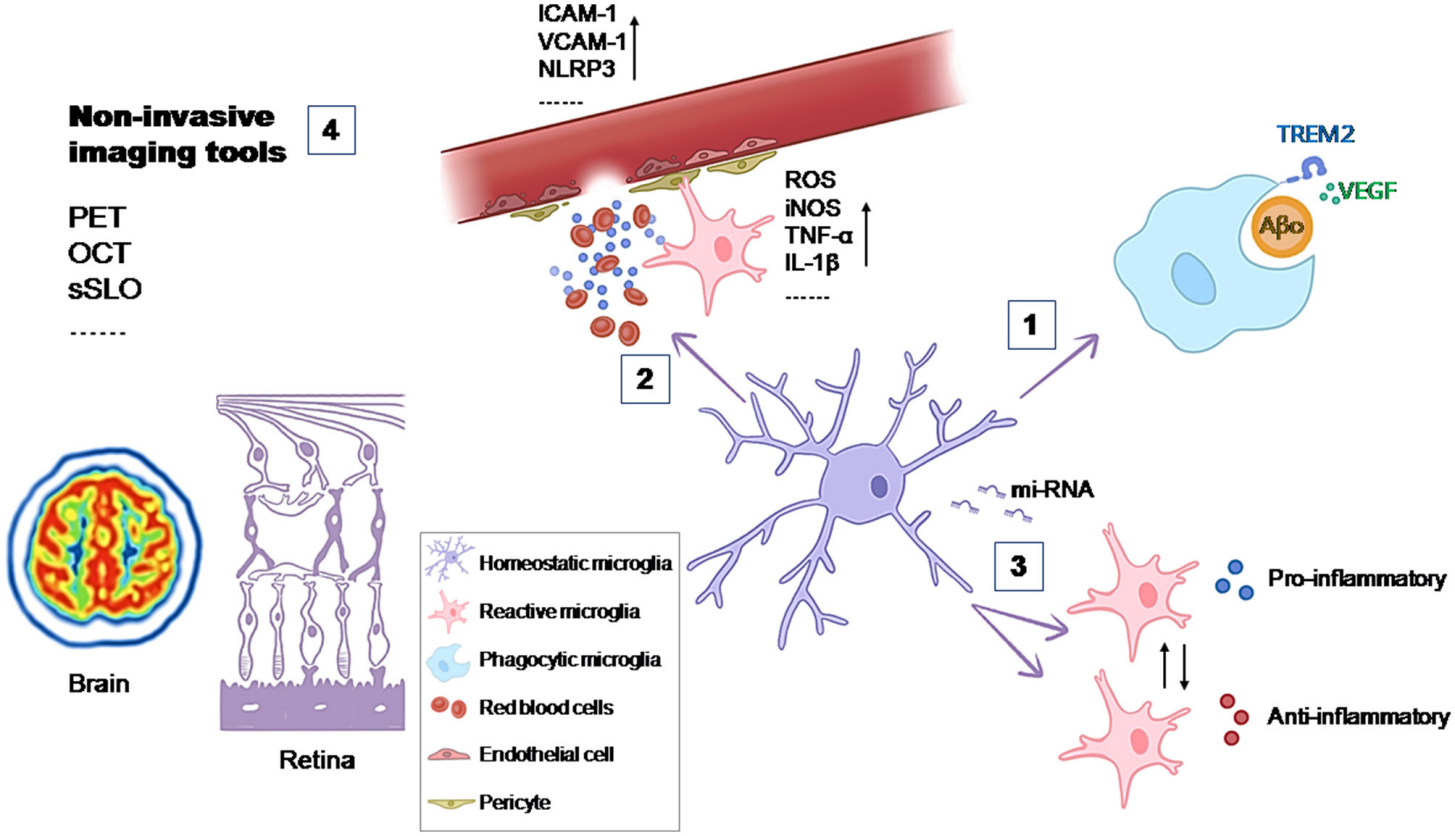
Contributions of microglia to the development of the disease and possible therapeutic targets. (1) The phagocytic activity of microglia is essential in maintaining tissue homeostasis. VEGF signaling increases the phagocytic function of microglia for Aβ oligomers (de Gea et al.); (2) The entry of substances from the peripheral blood into the CNS can active microglia and lead to oxidative stress and inflammation in the CNS (Mayer and Fischer); (3) Dysregulated miRNA expression in microglia contributes to disease progression by affecting microglial activation, cytokine production, and phagocytic activity (Jadhav), (4) Non-invasive imaging tools woluld allow to study microglia *in vivo* and increase the understanding of their contribution to the development of the disease (Etebar et al.).

The objective of this Research Topic was to explore the *Microglia in neurodegenerative diseases*, from their behaviors and roles in disease development to novel strategies for regulating their activity (Figure 1). Four contributions were gathered, including an original research, a review, and two mini reviews.

One important role of microglia in maintaining tissue homeostasis under physiological conditions is due to their phagocytic activity. Microglia can remove ineffective synapses, dying cells, and cellular debris. In the context of AD, microglia are suggested to clear amyloid-β (Aβ) plaques. When this function is impaired, microglia contribute to plaque formation. In an effort to enhance this microglial function, de Gea et al. reported about the importance of the Vascular Endothelial Growth Factor (VEGF) signaling. In *in vitro* and *ex vivo* AD models they observed that this signaling increases the phagocytic function of microglia for Aβ oligomers but not for fibrils. Furthermore, they also described that the activity of a protease induced by VEGF (ADAM) may be involved in Triggering Receptor Expressed on Myeloid cells 2 (TREM2)-dependent regulation of phagocytosis of Aβ oligomers (de Gea et al.).

Microglia not only respond to challenges within the CNS but also to systemic challenges, acting as major contributors to oxidative stress and inflammation in the CNS. The blood-brain barrier (BBB) is essential for preserving brain homeostasis by selectively blocking the entry of substances from the peripheral blood into the CNS. In their review for this Research Topic, Mayer and Fischer explored the intricate interactions at the BBB among microglia, endothelial cells, and other vascular elements, especially in the scenarios of stroke, diabetes, and systemic inflammation, where oxidative stress and inflammation are prevalent factors. Given that chronic neuroinflammation contributes to the development and progression of neurodegenerative diseases and subsequent neuronal damage, it is evident that the role of the BBB and its interaction with microglia is crucial for creating new therapeutic strategies to mitigate the harmful effects of inflammation in various neurodegenerative conditions.

Within the intense research focused on regulating microglial function in pathology, recent studies have emphasized the importance of small non-coding RNA molecules (miRNAs). In neurodegenerative diseases such as AD, PD, ALS, and Multiple Sclerosis (MS), dysregulated miRNA expression in microglia contributes to disease progression by affecting gene expression, modulating cytokine responses, and influencing phagocytosis. Jadhav's review summarizes the current knowledge on how miRNAs influence these processes. It also explores the implications of miRNA dysregulation in the transition of microglia from a neuroprotective to a neurotoxic phenotype, a pivotal factor in the progression of neurodegenerative diseases. In the review, specific miRNAs are also highlighted for their roles in modulating microglial responses during neuroinflammation and neurodegeneration in different neurodegenerative diseases. Emerging research on these small molecules offers valuable insights into the molecular mechanisms that govern microglial behavior, highlighting potential targets for modulating neuroinflammatory responses in disorders like AD, PD, ALS, and MS (Jadhav).

Understanding how microglia contribute to pathological conditions is essential for developing therapeutic strategies. Studying microglia *in vivo* could increase the understanding of their contribution and open new possibilities for diagnostic biomarkers. An overview of the current brain and retinal imaging tools (Positron emission tomography, optical coherence tomography, confocal laser scanning ophthalmoscopy) and their limitations for studying microglia *in vivo* was contributed to this Research Topic by Etebar et al..

It is clear that one of the great challenges of current research to cure or reduce the progression of neurodegenerative diseases is understanding how microglia contribute to these conditions. Research included in this Research Topic aims to address this. Once the molecular mechanisms that can modulate microglial functions are identified, it could lead to the discovery of therapeutic strategies aimed at these that might significantly alter the course of the neurodegenerative diseases.

